# When neoliberals become activists: social crisis threats motivate ingroup and outgroup prosociality among neoliberals

**DOI:** 10.3389/fpsyg.2025.1677265

**Published:** 2025-11-07

**Authors:** Janine Stollberg, Franziska Koch, Eva Jonas

**Affiliations:** Department of Psychology, University of Salzburg, Salzburg, Austria

**Keywords:** neoliberal beliefs, prosocial behavior, collective action, existential threat, outgroup helping

## Abstract

**Introduction:**

People who support neoliberal beliefs (i.e., who believe in individual responsibility and meritocracy) are less motivated to collectively act against social inequality or help those in need. At the same time, economic and humanitarian crises put a spotlight on injustice and harm. They represent an existential threat to people and should thus motivate prosocial behavior in line with salient humanitarian values.

**Methods:**

We conducted two experimental studies. Study 1 (*N* = 175) made an economic crisis salient. Study 2 (*N* = 205), conducted four weeks after the outbreak of the Ukrainian war, made the associated humanitarian crisis salient.

**Results:**

We found raising people's awareness of a social crisis (compared to a non-threatening control condition) increased prosociality and solidarity-based collective action, even when this conflicted with participants' neoliberal beliefs. In Study 1, a salient economic threat (compared to prosperity) buffered the negative effect of neoliberalism on prosociality and system change. In Study 2, the salient (vs. not salient) consequences of the Ukrainian war, increased solidarity with Ukrainian refugees, thereby overruling the negative effects of neoliberalism. Mediational analysis suggested that the threat effects on solidarity with Ukrainians were due to increased outgroup identification following threat. In both studies, the effects of neoliberalism were independent of related constructs, such as political conservatism or social dominance orientation.

**Discussion:**

The results show that social crisis threats can make neoliberalists more flexible in applying their ideological beliefs. The findings are discussed in the context of group-based processes in response to threats.

## Introduction

Rising economic inequality and the humanitarian consequences of war and displacement pose challenges to neoliberal societies. They represent social crises because they threaten core social values—such as security, health, and solidarity—create uncertainty about future outcomes, and urge people to act (Boin and 't Hart, 2007. However, neoliberal beliefs, which emphasize individual responsibility and meritocracy, can inhibit collective action and prosocial behavior (Bettache et al., 2020; Dutt and Kohfeldt, 2019; Ginn et al., 2022).

Despite this, prosocial behaviors—such as donating to humanitarian causes or expressing solidarity with refugees—are often observed during acute crises, even in neoliberal contexts (Slovic et al., 2017; Smith et al., 2018). This raises a critical question: Can the salience of economic or humanitarian crises mitigate or even reverse the negative influence of neoliberal ideology on prosocial behavior?

Existential threat research suggests that this is the case. Threat perceptions tend to motivate collective behavior in line with salient norms and shared values (Fritsche et al., 2011; Jonas et al., 2008, 2014; Stollberg et al., 2017, 2024). However, it remains unclear whether this effect extends to individuals who strongly endorse neoliberal beliefs. To address this gap, we conducted two experimental studies to test whether confronting people with the consequences of an economic threat (Study 1) and a humanitarian threat (Study 2) can buffer the negative association between neoliberalism and prosociality.

### Neoliberal beliefs decrease support for social change and prosociality

#### Neoliberalism as a system-stabilizing ideology

Originally rooted in economic and political principles, neoliberalism has evolved into the prevailing ideology in industrialized societies (Brown, 2003; Harvey, 2007). Central to this worldview are the ideals of a free market economy, individual responsibility, and meritocracy. People who endorse neoliberalism believe that prosperity and progress can be achieved through individual responsibility, competition, and the downregulation of governmental interference. This belief system implies that everyone has equal opportunities to succeed through hard work (Bay-Cheng et al., 2015). As a result, neoliberal ideology can lead to the legitimization of social and economic inequality by portraying the system as inherently fair (Azevedo et al., 2019; Bettache et al., 2020). Empirical evidence supports this notion at both individual and societal levels. Societies with strong neoliberal ideologies, that is, a high collective belief in meritocracy and economic freedom, are also characterized by high power distance (i.e., the acceptance of unequal hierarchical structures and power distributions), independent of objective inequality measured by the GINI index (Bettache et al., 2020). Similarly, individuals who strongly endorse neoliberal beliefs are more likely to justify the system and less likely to support collective efforts to challenge inequality (Girerd and Bonnot, 2020; Girerd et al., 2020). For instance, neoliberal women identified less as feminists and showed less support for collective action to reduce gender-based inequality (Girerd and Bonnot, 2020). In a similar vein, people with high neoliberal beliefs reported lower political identification, which predicted reduced support for the Yellow Vest Movement, a system-challenging political movement in France (Girerd et al., 2020).

Thus, neoliberal ideology represents a normative set of shared values and beliefs, grounded in individual freedom and meritocratic ideals. It is associated with the legitimization of inequality and opposition to social change that is related but different from other system-stabilizing ideologies, such as social dominance orientation (SDO, Pratto et al., 1994), and political conservatism (Azevedo et al., 2019; Bay-Cheng et al., 2015; Beattie et al., 2019).

#### Neoliberalism reduces prosociality

Neoliberal beliefs can be a barrier to social change when the system is perceived as just. Still, they can also undermine solidarity with those in need when identification with others is lacking. According to neoliberal ideology, individuals are responsible for their success and failure, which comes at the expense of group solidarity. High neoliberal beliefs are accompanied by greater self-interest (Beattie et al., 2019) and less concern for the collective (Nafstad et al., 2009). These beliefs are associated with less empathy and perspective-taking, in turn predicted reduced charitable giving to marginalized groups (Ginn et al., 2022). This lack of prosociality can extend to negative attitudes toward disadvantaged groups. For instance, Japanese neoliberals expressed more anti-immigrant attitudes (Igarashi and Ono, 2022), and US-American neoliberals showed less support for Central American asylum seekers (Dutt and Kohfeldt, 2019).

This tendency to disengage from helping indigent people may be explained by a lack of inclusion into one's ingroup. High neoliberals were less willing to help asylum seekers, whom they perceived as morally incompatible with their ingroup (Dutt and Kohfeldt, 2019). Together, these findings suggest that neoliberalism's endorsement of systemic inequality also manifests in reduced humanitarian concern, resulting in decreased prosocial behavior and reduced helping both within and outside the system. However, situational factors, such as salient social crises, may challenge the negative link between neoliberalism and prosociality because they highlight how shared values of equality and prosociality are violated.

### Salient social crises can motivate collective prosociality

Making people aware of a social crisis that challenges the values of equality, justice, and humanity can motivate prosocial and solidarity-based action, as it connects individuals to those in need. For humanitarian crises, it has been demonstrated that individuals who are not directly affected by the crisis become more prosocial when the violation of humanitarian values is salient. During the Syrian refugee crisis in 2015, people donated more money to the Red Cross refugee help program after the picture of the little refugee Alan Kurdi lying dead on the beach was published (Slovic et al., 2017). Moreover, tweeting about Alan Kurdi's death was associated with expressions of solidarity on social media 10 weeks later (Smith et al., 2018).

Comparable effects have been found for solidarity-based action that aimed at improving the rights of disadvantaged outgroup members. For instance, when members of an advantaged group perceived an outgroup as being unjustly treated, they engaged on behalf of the disadvantaged. They fought for their rights when they felt an emotional bond to those in need (Saab et al., 2015), when they identified with the outgroup (Klavina and van Zomeren, 2018), or when they shared a common identity (Subašić et al., 2011). Thus, salient social crises, in which shared values of equality and prosociality are at stake, can motivate solidarity with ingroup and outgroup members by increasing group ties.

### Threat perceptions motivate prosociality in line with salient social values

One possible explanation as to why salient social crises motivate prosocial behavior is that they are perceived as existential threats (Fritsche and Jugert, 2017). This is because threat perceptions motivate group-based responses and compliance with shared values that are activated in the situation (Fritsche et al., 2011). In other words, increased prosociality toward other group members when prosociality is the central value can be understood as a motivated threat response. Existential threat research has shown that when people are confronted with a situation that deprives them of their fundamental needs, the defense of salient values and group norms increases (Jonas and Fritsche, 2013). For instance, reminders of mortality can increase charitable giving when fairness or prosocial norms are salient (Jonas et al., 2008, 2013). Similar effects have been observed in response to threats to personal control. Individuals experiencing a loss of control were more likely to conform to group norms and even support social change when such norms were approved by their ingroup (Stollberg et al., 2017, 2024). Therefore, salient group norms and shared values should also become more important in the context of economic and humanitarian crises, acting as a guide to action, as these crises represent a threat to the basic needs of certainty and control (Fritsche and Jugert, 2017; Fritsche et al., 2011).

This leads to our central research question: How do salient crisis threats that emphasize shared values of equality and humanity interact with people's endorsement of neoliberal beliefs to promote prosociality and system change?

### The present research

Neoliberal ideology is grounded in meritocratic principles that hold individuals accountable for their outcomes across various life domains. The present research explores how this dispositional belief system interacts with situational crisis threats to influence prosocial behavior. Using an experimental approach, we examined whether making an economic threat (i.e., illustrating the negative consequences of increasing unemployment rates, even among the high-educated, for life and well-being in Study (1) or a humanitarian threat (i.e., displaying the negative consequences of the Russian war on Ukrainians 4 weeks after the outbreak in Study (2) salient would affect prosocial intentions among individuals with strong neoliberal beliefs.

We hypothesized that individuals endorsing neoliberal beliefs would show lower levels of prosociality (i.e., donations to aid agencies and help for refugees) and less support for system change (i.e., collective action and system justification) (correlation hypothesis). However, we expected that making an economic or humanitarian crisis salient, in which shared values of equality and humanity were violated buffer the negative effect of neoliberalism on prosociality (moderation hypothesis). This is because the threat of an economic or humanitarian crisis should connect people more to ingroup and outgroup members in need and increase prosociality in line with salient humanitarian values. Thus, we further predicted that increased identification with those in need would mediate the relationship between threat salience and prosociality (mediation hypothesis). While the correlation and moderation hypotheses were tested in both studies, the mediation hypothesis was only tested in Study 2.

### Transparency and openness

For each study, we report how we determined our sample size, all data exclusions (if any), all manipulations, and all measures in the study. All data, syntax code, and research materials are available at https://osf.io/qnfrt/?view_only=e3e2bfcf345b4cabab6e42446ecd03a4. The hypotheses, design, and analysis plan were not preregistered for Studies 1 and 2. The ethics board of our research institution approved all studies.

## Study 1

In Study 1, we tested for the impact of neoliberal beliefs and economic threat salience on prosociality. To manipulate threat salience, half of the participants were exposed to a scenario highlighting the anticipated decline in prosperity and social equality for young academics. This violated the shared value of equality and fairness for their ingroup. The other half read a non-threatening scenario in which prosperity remained attainable. As dependent variables, we measured participants' willingness to donate to different Aid agencies as well as their support for collective action to reduce social inequality, and their justification of the current system. We hypothesized that neoliberal beliefs would negatively predict all forms of prosociality. Furthermore, we expected that making the economic threat salient would buffer the negative relationship between neoliberal beliefs and prosocial behavior.

### Method

#### Participants and design

The study was conducted as an online experiment on attitudes toward social and economic inequality. The participants were recruited through the local university's recruitment system and via digital advertisements. One hundred eighty-three participants completed the study. Five participants were excluded because they were not convinced by the economic threat manipulation, and three were excluded because they did not indicate their age (according to our ethical approval, we had to ensure the age of majority for all participants), resulting in a final sample of *N* = 175. The participants were *M* = 25.30, *SD* = 10.06 years old, with 122 identifying as female and 53 as male, and none as divers. 5.1% held a lower secondary level of education, 80.6% an upper secondary level (e.g., high-school degree or vocational training), and 14.3% had a tertiary education (e.g., bachelor's or master's degree). All participants had a European nationality, with 49.1% Germany and 48.6% Austria representing the largest groups.

The study had a between-subjects design with one experimental factor with two levels, making the economic threat salient or not. The sample size was determined by a priori power analysis to detect a small-sized moderation effect, f^2^ = 0.05, in a linear multiple regression, with a statistical power of 80% and an error probability of 0.05. The analysis revealed a required sample size of *N* = 159. We aimed to recruit approximately 10% more participants to account for potential exclusions.

#### Procedure

After giving informed consent, participants completed demographic questions (age, gender, nationality, education) and indicated their political orientation using a slider from 1 (left) to 10 (right) (Breyer, 2015). Then, their generalized self-efficacy and spirituality were assessed^1^, before they were randomly assigned to the economic threat salient or non-salient conditions. Afterwards, the dependent variables —collective action intentions, donation intentions, and system justification—were assessed together with participants' neoliberal beliefs. The presentation of the measures was block-wise counterbalanced, with either collective action and donation coming first and system justification and neoliberal beliefs coming last, or vice versa. Then, the participants were thanked, debriefed, and informed about the fictitious nature of the newspaper article. Psychology students received course credit for participation.

#### Manipulation of economic threat salience

In the economic threat salient condition, participants read a fictitious newspaper article to make economic inequality and the violation of humanitarian values salient to participants. The article presented arguments and a graph illustrating that the gap between the rich and the poor is widening, also in wealthy European countries. It was stated that even well-educated people with academic degrees will face a decrease in prosperity and uncertainties in finding employment. This was illustrated by an example of a family father who, despite being employed, is no longer able to provide for his family with his wages and is dependent on help. In contrast, in the control condition, no economic threat or inequality was made salient. The participants read a fictitious newspaper article of similar length. The article was illustrated with an example and a graph that presented a positive picture of economic development in wealthy European countries, showing a decline in the unemployment rate. Then, in both conditions, participants were asked to briefly describe how they felt after reading the article and how the issue related to their personal lives, to ensure that participants became engaged with the topic. No further manipulation check was included.

#### Measures

##### Willingness to donate to Médecins Sans Frontières and Oxfam

We measured participants' willingness to donate to two different Aid organizations, Médecins Sans Frontières (MSF) and Oxfam, with two items each, measuring attitudinal and behavioral support. After describing the aim of each Aid Organization, the participants indicated whether they find the organization “*worthy of support in general*”, and “*how often would you be willing to donate to the organization*” on a 10-point scale, ranging from 1 (= not at all) to 10 (= absolutely/regularly). We combined both items into a scale to measure willingness to donate to each organization because attitudinal and behavioral support are both expressions of prosociality following threat (Jonas et al., 2002). Their correlation was sufficiently high, for MSF, *r*(173)=0.47, and for Oxfam, *r(*173) =0.61. Since MSF is an Aid Organization providing medical and humanitarian aid in global crisis regions, whereas Oxfam campaigns against poverty and social inequality, we considered willingness to donate to each organization as two separate dependent variables.

##### Collective action against inequality

We measured participants' support for collective action that targets social and economic inequality. Therefore, the participants answered five items on a 7-point scale (1 = very unlikely to 7 = very likely), such as, “*How likely is it that you will: …participate in a demonstration against social inequality”, “…sign a petition against social inequality”*, adapted from Mikołajczak and Becker (2019). We built the mean score across all five items to assess participants' willingness to support systemic change, α =0.83. This was followed by four items measuring perceived collective efficacy to tackle social and economic injustice effectively. This measure was included for exploratory purposes and is not considered in the results section.

##### System justification

We measured participants' perceptions of the social system as fair and just with the System Justification Scale adopted from Kay and Jost (2003), on a 7-point scale (1 = absolutely disagree to 7 = absolutely agree). The scale consisted of eight items, two reverse-coded, such as “*Most political decisions are for the good of everyone.”, “All people have a fair chance to achieve happiness and prosperity.”*, α =0.79.

##### Neoliberal beliefs

To assess participants' neoliberal beliefs, we used eight items from the Neoliberal Beliefs Inventory (Bay-Cheng et al., 2015), α = 0.83. Participants indicated their agreement with assumptions, assessing the neoliberal sub facets ”personal wherewithal”, “competition”, “system inequality”, and “government interference”, with one to three items each, such as, “*People who complain about social inequality often just blame others for their problems.”, “Every goal is achievable with enough hard work and willpower.”, “Your success in life is determined more by your effort and less by the social system.”, “Social programs of the state create false incentives and offer unearned rewards.”*, on a 5- point scale (1 = absolutely disagree to 5 = absolutely agree).

##### Other variables

Generalized self-efficacy (Schwarzer and Jerusalem, 1995), α = 0.83, and intrinsic spirituality, with seven items, α = 0.98, adapted from (Hodge, 2003).

### Results

To test whether neoliberalism is associated with less prosociality, we ran a correlation analysis across conditions (see Table 1). As expected, neoliberal beliefs correlated significantly negatively with willingness to donate and intentions to collectively act against social inequality, and significantly positively with system justification. Prosociality and support for system change did not differ between the economic threat salient conditions.

**Table 1:** Descriptives and correlations for neoliberal beliefs, prosociality, support for system change, system justification, political orientation, general self-efficacy, and spirituality, depending on economic threat salience in study 1.

**Variable**	**Economic threat salient (*****n*** = **93)**		**No threat salient (*****n*** = **82)**				***r(173)***					
	***M***	***SD***	***M***	***SD***	***t*** **(173)**	**1**	**2**	**3**	**4**	**5**	**6**	**7**
1. Donation (MSF) *Scale range 1–10*	6.96	1.72	6.95	2.00	−0.02							
2. Donation (Oxfam) *Scale range 1–10*	5.90	1.96	6.18	2.40	0.84	0.68^***^						
3. Collective action *Scale range 1–7*	4.20	1.11	4.51	1.49	1.55	0.21^**^	0.31^***^					
4. System justification *Scale range 1–7*	3.26	0.95	3.52	0.93	1.85	0.06	−0.11	−0.29^***^				
5. Neoliberal beliefs *Scale range 1–5*	2.62	0.75	2.58	0.80	−0.43	−0.19^*^	−0.35^***^	−0.51^***^	0.15^*^			
6. Political orientation *Scale range 1–10*	3.74	1.47	3.41	1.55	−1.43	−0.20^**^	−0.33^***^	−0.47^***^	0.02	0.49^***^		
7. General self-efficacy *Scale range 1–4*	2.88	0.42	2.99	0.45	−1.59	−0.17^*^	−0.09	−0.16^*^	0.11	0.37^**^	0.15^*^	
8. Spirituality *Scale range 1–7*	2,89	1.67	2.83	1.64	0.24	0.01	−0.12	−0.004	−0.14	0.15^*^	0.09	0.09

Asterisks indicate p-values: ^*^<*p* = 0.05, ^**^<*p* = 0.01, ^***^<*p* = 0.001.

#### Moderation analyses

To test our main hypothesis that economic threat salience buffers the effect of neoliberal beliefs on prosociality and system change, we conducted four separate regression-based moderation analyses, using the *process script*, version 4.2 for SPSS (Hayes, 2017). Neoliberal beliefs were entered as the predictor, economic threat salience as moderator, dummy coded, and the willingness to donate to MSF, Oxfam, support for collective action, and system justification as dependent variables, respectively. Age, gender, political orientation, generalized self-efficacy, and spirituality were entered as covariates to all moderation analyses.

##### Willingness to donate

**Figure 1:**
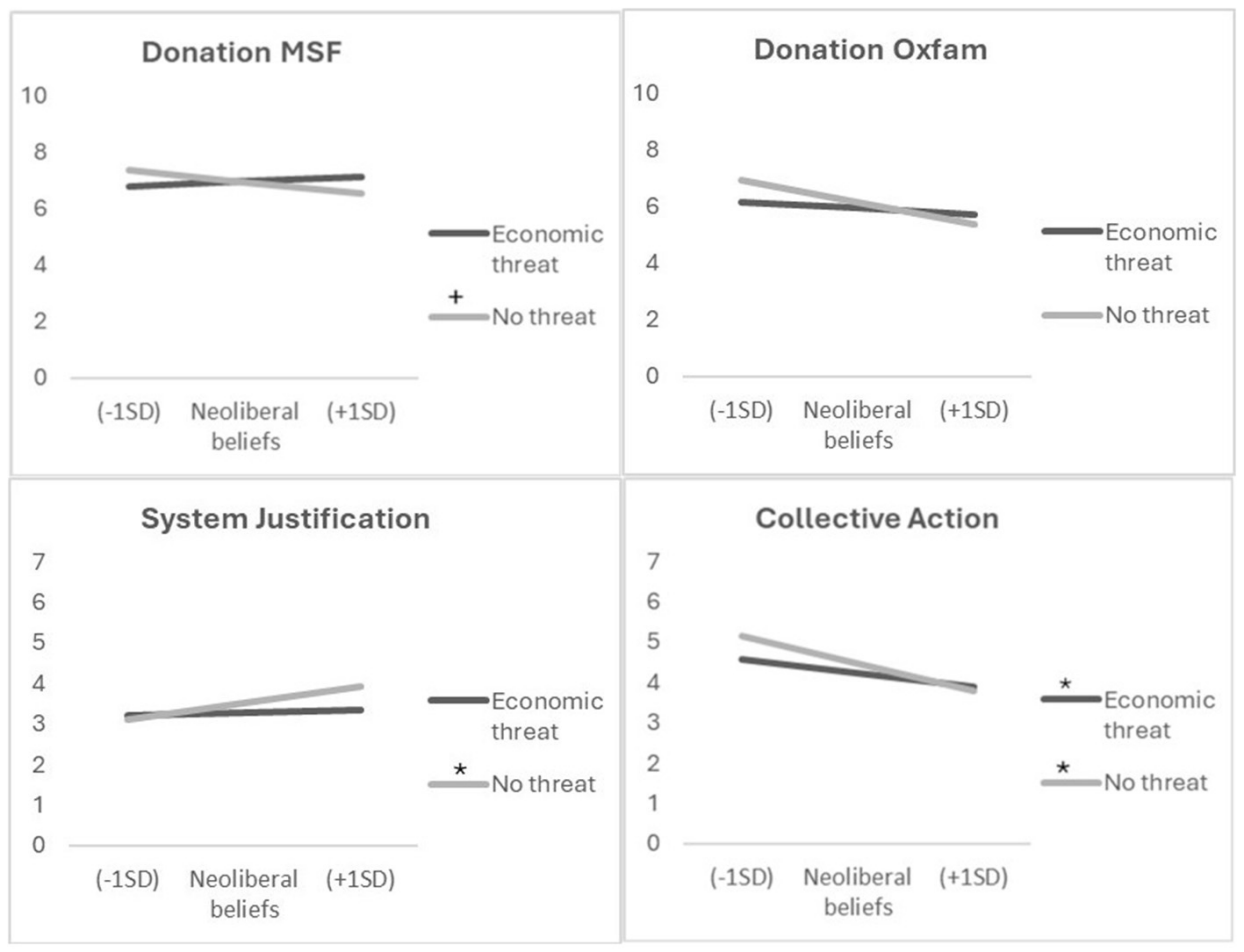
Results of the moderation analyses in study 1: economic threat salience buffers the negative effect of neoliberal beliefs on prosociality. Slopes are marked by * = *p* < 0.01, and + = *p* < 0.10.

The moderation analyses showed a negative relation between neoliberal beliefs and willingness to donate to Oxfam, *b* = −0.99, *SE* = 0.32, *t*(166) = −3.09, *p* = 0.002, 95% CI [−1.61, −0.36], and a non-significant trend to donate less to MSF, *b* = −0.52, *SE* = 0.29, *t*(166) = −1.80, *p* = 0.074, 95% CI [−1.10, 0.05]. Economic threat salience affected this relationship as predicted, showing a significant moderation for donation to MSF, *b* = 0.73, *SE* = 0.36, *t*(166) = 2.04, *p* = 0.043, 95% CI [0.02, 1.45] and a nonsignificant tendency for donation to Oxfam, *b* = 0.70, *SE* = 0.39, *t*(166) = 1.79, *p* = 0.075, 95% CI [−0.07, 1.48]. Simple slope analyses for donation to MSF revealed a buffering effect of economic threat salience: Neoliberal beliefs did not predict willingness to donates when economic threat was salient, *b* = 0.21, *SE* = 0.28, *t*(166) = 0.76, *p* = 0.447, 95% CI [−0.34, 0.76] but showed a non-significant tendency to decrease the willingness to donate to MSF when participants were not reminded of economic inequalities, *b* = −0.52, *SE* = 0.29, *t*(166) = −1.80, *p* = 0.074, 95% CI [−1.10, 0.05], (see Figure 1). The model explained 9,7% of the variance in willingness to donate for MSF, and 22,18% of the variance in willingness to donate for Oxfam.

##### Support for system change

Two separate moderation analyses, with collective action against inequality and system justification as dependent variables, revealed a similar pattern. While neoliberal beliefs were significantly negatively related to collective action, *b* = −0.88, SE = 0.17, *t*(166) = −5.11, *p* < 0.001, 95% CI [−1.22, −0.54], and positively to system justification, *b* = 0.54, SE = 0.14, *t*(166) = 3.72, *p* < 0.001, 95% CI [0.25, 0.82], again, this relation was affected by economic threat salience, *b* = 0.44, SE = 0.21, *t*(166) = 2.07, *p* = 0.040, 95% CI [0.02, 0.86] for collective action, and *b* = −0.46, SE = 0.18, *t*(166) = −2.60, *p* = 0.010, 95% CI [−0.82, −0.11] for system justification. Simple slope analyses showed that economic threat salience eliminated the association between neoliberal beliefs and system justification when economic threat was salient, b = 0.07, SE = 0.14, *t*(166) = 0.54, *p* = 0.591, 95% CI [−0.20, 0.34], but not when it was not, *b* = 0.54, SE = 0.14, *t*(166) = 3.72, *p* < 0.001, 95% CI [0.25, 0.82]. While participants with neoliberal beliefs did no longer justify the system, when economic threat was salient, they still showed less support for collective action to address the inequality, *b* = −0.44, SE = 0.16, *t*(166) = −2.71, p = 0.008, 95% CI [−0.76, −0.12], but to a lesser degree than when economic threat was not salient, *b* = −0.88, SE = 0.17, *t*(166) = −5.11, *p* < 0.001, 95% CI [−1.22, −0.54], (see Figure 1). The model explained 37,84% of the variance in collective action, and 15,61% of the variance in system justification.

#### Discussion

We found that neoliberal beliefs were associated with less prosociality and a lower willingness to change the system, supporting previous findings (Azevedo et al., 2019; Dutt and Kohfeldt, 2019; Ginn et al., 2022; Girerd and Bonnot, 2020; Girerd et al., 2020). However, when participants were confronted with an economic threat that violated shared values of equality and fairness for their ingroup, the negative effects of neoliberalism on prosociality were attenuated. Specifically, economic threat salience buffered the association between neoliberalism and both the willingness to donate to MSF and the justification of the current system as fair. Threat salience also weakened the negative relationship between neoliberalism and collective action, although it did not significantly influence the willingness to donate to Oxfam. In other words, people with high neoliberal beliefs differed less from people with low neoliberal beliefs in prosociality when an economic threat was salient.

However, the buffering effects of economic threat salience varied by type of prosocial behavior. While economic threat salience completely buffered the negative effects of neoliberalism on prosociality, specifically in the context of humanitarian aid (i.e., donating to MSF), neoliberalism's effects on active system change were less mitigated. Neoliberals still showed reduced support for collective action or spending less money for groups that aim to change economic inequality, such as Oxfam. In summary, the results provide initial evidence that neoliberals exhibit more prosocial behavior in line with salient humanitarian values when confronted with the negative consequences of the economic crisis. They suggest that neoliberals become more prosocial when threat is salient, and that situational threats to humanitarian and equalitarian values can undermine the effect of ideological convictions. To test this prediction more directly for prosociality toward outgroup members, we set up Study 2, where we explicitly made a humanitarian threat salient: the consequences of the Russian invasion for Ukrainians.

## Study 2

Study 2 was set up as a conceptual replication for the context of a humanitarian crisis, explicitly focusing on prosociality toward outgroup members. Participants were randomly assigned to view either a video highlighting the humanitarian consequences of the Russian invasion of Ukraine or a neutral, non-threatening control video. We expected neoliberal beliefs to negatively predict prosociality (i.e., helping Ukrainian refugees and protesting against the war) and a salient humanitarian threat to buffer this effect. As we expected threat salience to connect people to those in need, which should, in turn, promote prosociality in line with salient humanitarian values, we tested whether increased identification with Ukrainians mediated the effects of threat on prosociality (mediation hypothesis). In addition, we controlled for the impact of social dominance orientation (SDO; Pratto et al., 1994) on prosociality, as a related but distinct system-stabilizing ideology to neoliberalism.

### Method

#### Participants and design

The study was conducted as an online experiment on “Perceptions of current media content”. The participants were recruited through the local university recruitment system, via digital advertisements, and on SurveyCircle.com. Two hundred twenty-nine participants completed the study. Twenty-four participants were excluded because they failed the attention check, resulting in a final sample of *N* = 205. The participants were *M* = 29.07, *SD* = 12.47 years old, 119 identified as female, 85 as male, and one as diverse. 8.8% held a lower secondary level of education, 62.9% an upper secondary level (e.g., high-school degree or vocational training), and 28.3% had a tertiary education (e.g., bachelor's or master's degree). All participants had a European nationality or lived in a European country, with 88.3% Germany and 7.8% Austria representing the largest groups.

The study had a between-subjects design with one experimental factor with two levels, making the consequences of the Ukrainian war salient or not. Based on the results from Study 1, sample size was determined by a priori power analysis to detect two small-sized effects, f^2^ = 0.05, for neoliberalism and for the threat by neoliberalism interaction in a linear multiple regression, with a statistical power of 80% and an error probability of 5%. The analysis revealed a required sample size of *N* = 196. We aimed at recruiting a 10% larger sample to account for possible exclusions.

#### Procedure

After providing informed consent, participants completed demographic questions and indicated their political orientation, identical to those in Study 1. Then, the ideological orientations of neoliberalism and social dominance orientation were assessed, followed by a measure of utopianism, before participants were randomly assigned to either the humanitarian threat salient (i.e., the Ukrainian war) or non-salient conditions. After reporting their actual affective states, the participants indicated their willingness to support Ukrainians, to justify the social system, and their identification with different groups (i.e., Ukraine, Russia, Humanity). The presentation of the dependent measures of solidarity and system justification was counterbalanced. Then, the participants were thanked and debriefed, and psychology students received course credit for participation.

#### Ideological orientations

##### Social dominance orientation

We measured participants' social dominance orientation using eight items adapted from Ho et al. (2015), a 7-point scale (1 = very unlikely to 7 = very likely), α =0.80. Example items are, “*Some groups of people are simply inferior to other groups*”, “*Group equality should not be our primary goal*.”

##### Neoliberal beliefs

To assess participants' neoliberal beliefs, participants responded tothe same eight items from the Neoliberal Beliefs Inventory (Bay-Cheng et al., 2015) as in Study 1, on a 5-point scale (1 = absolutely disagree to 5 = absolutely agree), α = 0.84.

##### Manipulation of humanitarian threat salience

In the humanitarian threat salient condition, participants viewed a two-and-a-half-minute video about the war in Ukraine and its consequences, based on combined excerpts from actual news reports (video available at https://osf.io/qnfrt/?view_only=e3e2bfcf345b4cabab6e42446ecd03a4). At the beginning of the video, they read “24.02.22 Russia started a war against Ukraine”. This was followed by images of destroyed cities in Ukraine, people fleeing, and peace demonstrations, accompanied by the sound of explosions. Excerpts from reports and news items about the social, economic, and political consequences of the war were also shown. This was followed by further images of the destruction and humanitarian suffering of the people in Ukraine. In contrast, in the control condition, no humanitarian threat or values were made salient. The participants viewed a video entitled “Busy as a bee?” (Volto and Friren, 2018), which explored the question of whether bees and ants are better workers than humans, of similar length.

Then, in both conditions, participants reported how the video made them feel on 26 items measuring state affect for fear with eight items, such as horrified, terrified, shocked (adapted from Franchina et al., 2024), eight items for anxiety, such as worried, nervous, afraid (Stollberg et al., 2024), and 10 items for positive activation (PA Scale from the German version of the PANAS, Breyer and Bluemke, 2016), on a 5-point scale (1 = not at all to 5 = extremely). Participants in the humanitarian threat salient condition were more anxious, *M* = 2.65 (*SD* = 1.00), and experienced more fear *M* = 2.87 (*SD* = 1.04) than participants in the neutral control condition, *M* = 1.40, *SD* = 0.52, *t*(203) = −11.42*, p* < 0.001 for anxiety, and *M* = 1.31, *SD* = 0.47, *t*(203) = −14.07*, p* < 0.001 for fear, indicating that our threat manipulation was successful.

#### Dependent measures

##### Solidarity

We measured participants' support of Ukrainians with nine items on a 7-point scale (1 = absolutely disagree to 7 = absolutely agree). Five items assessed participants' willingness to demonstrate solidarity with Ukrainians, adapted from Mikołajczak and Becker (2019), such as “*How likely is it that you will participate in societal actions against the war in Ukraine, such as: “joining a demonstration”, “signing a petition”, “post social media content”*. Additional four items assessed participants' willingness to “*donate money*”, “*donate clothes*”, and “*help refugees*”, α = 0.83.

##### System justification and values

System justification was assessed with the short system justification scale, consisting of eight items, on a 7-point scale (1 = absolutely disagree to 7 = absolutely agree), as used previously in Study 1 (Kay and Jost, 2003), α =0.78. For exploratory purposes, we also measured participants' perceived personal importance of the values universalism, sociality, tradition, and security as a trait (Boer, 2014).

##### Identification with Ukraine

To assess identification with the outgroup of Ukrainians, participants reported their agreement with five items, reflecting the emotional connection participants experienced, adopted from Leach et al. (2008) on a 7-point scale (1 = does not apply at all to 7 = fully applies), α = 0.80. Example items are, “*I feel a bond with Ukrainians*”, “*I feel solidarity with Ukrainians*”, “*I feel committed to Ukrainians*.”

##### Other variables

Identification with a global identity, identification with Russia, anger toward Russia, and utopianism were included for exploratory purposes and are not considered in the results section.

#### Results

In line with expectations, neoliberal beliefs, SDO, and political orientation were associated with lower levels of solidarity, and SDO with more system justification (see Table 2).

**Table 2:** Descriptives and correlations for neoliberal beliefs, solidarity, system justification, social dominance orientation, and political orientation in study 2.

**Variable**	**Humanitarian threat salient (*****n*** = **98)**		**No threat salient (*****n*** = **107)**		***r*** **(203)**			
	***M***	***SD***	***M***	***SD***	**1**	**2**	**3**	**4**
1. Solidarity	4.99	1.13	4.72	1.10				
2. System justification	3.63	0.90	3.60	0.98	−0.05			
3. Neoliberal beliefs	3.60	1.17	3.46	1.05	−0.43^***^	0.13		
4. SDO	2.79	1.01	2.57	0.99	−0.40^***^	0.27^***^	0.48^***^	
5. Political orientation	4.07	1.73	3.98	1.56	−0.45^***^	0.10	0.51^***^	0.47^***^

Asterisks indicate *p*-values: ^*^<*p* = 0.05, ^**^<*p* = 0.01, ^***^<*p* = 0.001.

##### Moderation analyses for solidarity and system justification

We conducted two separate moderation analyses, with solidarity and system justification as dependent variables, with neoliberal beliefs as the predictor, humanitarian threat salience as a moderator (dummy coded), and SDO, political orientation, gender, and age as covariates. While neoliberal beliefs, SDO and a more right political orientation reduced solidarity with Ukrainians, humanitarian threat salience counteracted this effect for all participants (see Table 3). Threat salience increased solidarity with Ukrainians, independent of neoliberal beliefs, as no moderation could be observed. Together, demographic variables, ideological beliefs and threat salience independently explained 34% of the variance in solidarity with the outgroup of Ukrainians.

**Table 3:** Results of the moderation analysis for solidarity as dependent variable in study 2.

**Variable**	***coeff***	***SE***	***t(197)***	***p***	***95% CI***	***total R^2^***
Constant	6.26	0.32	19.45	< 0.001	[5.63, 6.90]	0.34
Age	0.004	0.006	0.70	0.482	[−0.01, 0.01]	
Gender	−0.38	0.14	−2.80	0.005	[−0.65, −0.11]	
Political orientation	−0.15	0.05	−3.10	0.002	[−0.25, −0.06]	
SDO	−0.20	0.08	−2.55	0.012	[−0.35, −0.05]	
Neoliberal beliefs	−0.30	0.09	−3.26	0.001	[−0.49, −0.12]	
Threat salience	0.33	0.13	2.56	0.011	[0.08, 0.59]	
Threat x neoliberal beliefs	0.15	0.12	1.27	0.204	[−0.08, 0.38]	

Running the same moderation analysis for system justification as dependent variable revealed a positive association with SDO, *b* = 0.26, SE = 0.08, *t*(197) = 3.38, *p* < 0.001, 95% CI [0.11, 0.41], but no relation with neoliberal beliefs, *b* = −0.07, SE = 0.09, *t*(197) = −0.74, *p* = 0.461, 95% CI [−0.25, 0.11] or political orientation, *b* = −0.04, SE = 0.05, *t*(197) = −0.87, *p* = 0.386, 95% CI [−0.14, 0.05]. There was no threat, *b* = −0.03, SE = 0.13, *t*(197) = −0.20, *p* = 0.844, 95% CI [−0.28, 0.23], nor threat x neoliberal beliefs interaction effect, *b* = 0.15, SE = 0.12, *t*(197) = 1.26, *p* = 0.208, 95% CI [−0.08, 0.38]. The model explained 9% of the variance.

##### Identification with Ukrainians as a mediator

**Figure 2:**
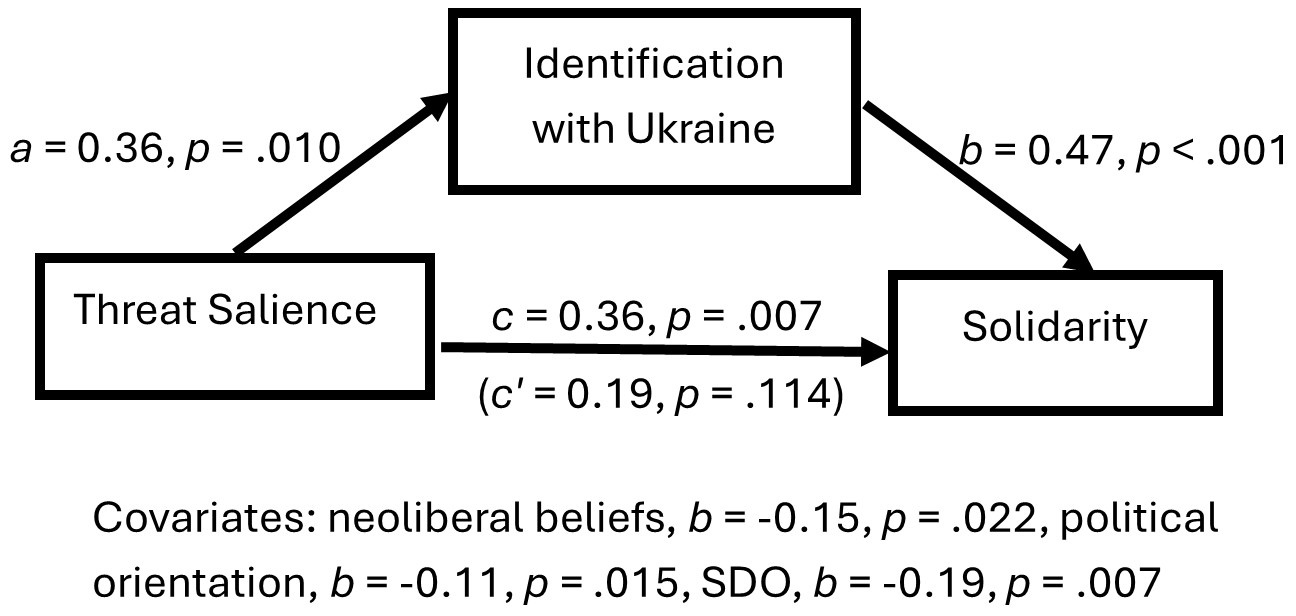
Humanitarian threat salience increased solidarity with Ukrainians through increased outgroup identification.

We expected threat salience to increase solidarity in line with the salient value of prosociality toward the Ukrainian outgroup through increased outgroup identification. Participants in the threat salient condition should show more willingness to help Ukrainians and protest against the war because they feel a stronger bond with the Ukrainian outgroup. To test this prediction, we conducted a mediation analysis with threat as the predictor (dummy-coded), identification with Ukrainians as the mediator, solidarity as the outcome, and neoliberal beliefs, social dominance orientation, and political orientation as covariates. The results showed a full mediation (see Figure 2). When the threat of the Ukrainian war was salient, participants identified more strongly with the outgroup of Ukrainians, which explained the increased solidarity, with an indirect effect of 95% CI [0.05, 0.32]. An exploratory moderated mediation analysis showed that threat salience effects on outgroup identification were not moderated by neoliberal beliefs (see Supplemental material).

#### Discussion

Our findings show that making the humanitarian consequences of the Russian attack on Ukraine salient increased participants' willingness to express solidarity with Ukrainians and to support them through donations and aid. This effect of humanitarian threat salience emerged independently of participants' ideological beliefs, including neoliberalism, political conservatism, and social dominance orientation, which together explained nearly 30% of the variance in solidarity. However, the threat salience effect emerged for participants low and high in neoliberal beliefs, as no moderation could be observed.

Notably, we found that increased outgroup identification mediated the effect of threat salience on solidarity. When the violation of humanitarian values was made salient, participants felt a stronger emotional connection to Ukrainians, which in turn predicted their willingness to support and help them. This suggests that situational cues can override ideological tendencies: While neoliberal beliefs usually decrease people's concern for the collective and empathy for others (Ginn et al., 2022; Nafstad et al., 2009), confronting people with a humanitarian crisis can foster emotional bonds and identification with those in need, thereby promoting prosocial behavior.

## General discussion

In two experimental studies, we found converging evidence that the salience of social crisis threats can attenuate the negative impact of neoliberal beliefs on prosociality. When no threat was salient, participants who endorsed neoliberal beliefs were less likely to engage in prosocial behavior. They reported less willingness to donate money to Aid Agencies, help Ukrainian refugees, support collective actions that target social inequality, and perceived the social system as fairer. However, when participants were confronted with an economic or humanitarian crisis threat that highlighted violations of shared values like equality and humanity, the negative effect of neoliberal beliefs on prosociality was buffered. Interestingly, the buffering effect of threat salience varied depending on the type of prosocial behavior. While threat salience eliminated the negative effect of neoliberal ideology on humanitarian outgroup help and solidarity in the context of both the economic and humanitarian crisis, the effect was weaker for collective action against inequality (Study 1). In Study 2, we found that increased identification with those in need mediated the effect of humanitarian threat salience on prosociality, suggesting that group processes play a key role in overriding ideological barriers. Overall, the present findings offer novel insights into how situational factors can counteract the influence of dispositional neoliberal beliefs on prosocial behavior.

### Neoliberal beliefs reduce different forms of prosociality

The present results indicate that neoliberal beliefs are associated with a lower willingness to change the system. This confirms previous research that has linked neoliberal ideology to system justification and the acceptance of inequality between the rich and the poor, as well as between women and men (Azevedo et al., 2019; Bay-Cheng et al., 2015; Ginn et al., 2022; Girerd and Bonnot, 2020; Girerd et al., 2020). We also found that neoliberal beliefs were associated with reduced solidarity toward those in need. High neoliberals donated less to Aid agencies and expressed less willingness to help Ukrainian refugees. This complements previous research showing that neoliberals express more prejudice toward immigrants (Dutt and Kohfeldt, 2019; Igarashi and Ono, 2022). However, our findings challenge these findings for the situation of threat.

### Threat salience buffers the negative impact of ideological beliefs on prosociality

Our results show that situational factors can moderate the adverse effects of neoliberalism on prosocial behavior. When economic or humanitarian threats were made salient, individuals with strong neoliberal beliefs exhibited greater flexibility in their prosocial responses. In other words, neoliberals no longer behaved like neoliberals when they were confronted with an economic threat, such as the growing gap between the rich and the poor, or a humanitarian threat, such as Russia's attack on Ukraine. These findings support the notion that highlighting violations of shared values like equality and humanity can buffer the negative influence of neoliberalism on prosociality.

Fritsche et al. (2011) interpret this flexible responding in the face of a social crisis threat as a motivated threat response. Studies that showed increased norm compliance following threat support this view. When individuals perceive a situation as threatening, they become more motivated to adapt and act in accordance with salient values and shared norms (Jonas et al., 2008; Stollberg et al., 2017, 2024). This was particularly the case when the salient values were shared by one's ingroup and when the norm was approved by fellow ingroup members (Stollberg et al., 2017, 2024). That is, people tend to cling to their ingroup in the face of a threat, and the ingroup becomes more important in general (Fritsche et al., 2017). These group-based processes may have contributed to our findings, helping to explain why neoliberals ceased to justify the system when confronted with an economic threat that violated their ingroup's values of fairness and equality (Study 1). Similarly, these processes may have mediated the effects of threat on outgroup helping behavior observed in Study 2.

### Prosociality as a group-based response to threat

#### Economic threat salience violated the ingroup norm of equality

In Study 1, we made an economic crisis salient, which threatened equal opportunities for young academics. Our manipulation illustrated that even highly educated and high-performing individuals were vulnerable to unemployment and financial instability. They were no longer able to move up the ladder because individual performance is no longer rewarded. This scenario likely challenged the neoliberal ingroup's belief that success is attainable through hard work and merit. From a neoliberal standpoint, such outcomes are perceived as unjust, thereby undermining the meritocratic norm. This aligns with our finding that neoliberals no longer defended the system when an economic threat was salient.

However, simply raising awareness of economic inequality may not be sufficient to motivate collective action among neoliberals. If inequality is perceived as the result of individual effort, it does not violate the meritocratic norm of the neoliberal ingroup and may not trigger threat perceptions and group-based action. Consequently, neoliberals may feel less connected to the collective because it is not necessary (i.e., the situation is individually fair), and experience less injustice and anger, which are central determinants of collective action (Agostini and van Zomeren, 2021). Future research should explore how different normative interpretations of inequality influence prosocial motivation and support for social change.

#### Humanitarian threat salience affected solidarity via identification with the outgroup

Our findings show that a salient humanitarian threat increased solidarity with the Ukrainian outgroup. Contrary to expectations, this effect was equally strong among low and high neoliberals. All participants felt threatened by the Ukrainian war and were motivated to act in solidarity with the outgroup.

Importantly, we found evidence for outgroup identification as a mediating process variable: Making people aware of the severe humanitarian consequences of the Ukrainian war motivated prosocial intentions through increased identification with the Ukrainian outgroup. This aligns with prior research demonstrating that identification with those in need is a key driver of solidarity-based action (Klavina and van Zomeren, 2018; Wolf et al., 2025). The salience of the crisis activated humanitarian values, fostering a sense of closeness to the outgroup. Salient humanitarian values may go hand in hand with a stronger communal relationship between one's own national ingroup and the outgroup of Ukrainians. A communal relationship is characterized by reciprocal helping and mutual support, increasing the emotional bond between groups (Fiske, 1992), thereby promoting outgroup identification (Klavina and van Zomeren, 2018). This aligns with the present finding that participants reported stronger emotional connections and commitments to Ukrainians, as measured by the outgroup identification measure, which in turn predicted greater prosociality. Moreover, it emphasizes the notion that threat salience can directly counteract the neoliberal tendency to exclude outgroups (Dutt and Kohfeldt, 2019). Alternatively, the crisis may have strengthened identification with a common ingroup identity of Europeans, which could also have contributed to increased solidarity-based action. Previous research has shown that salient norms of recategorization have been followed more rigorously in the face of threat (Giannakakis and Fritsche, 2011). Similarly, the impact of the Ukrainian war did not only confront participants with the suffering of the Ukrainian outgroup but could have activated a more inclusive ingroup of Europeans that is now threatened by the Russian outgroup. Future research could examine whether the present findings extend to non-European countries at war.

In addition to expanding the social self to include the outgroup in need, emotional processes may have been reinforced by the threat salience manipulation. Witnessing the humanitarian crisis in Ukraine may have moved participants and strengthened their emotional and social ties to Ukrainians. Participants may have experienced a sense of shared fate—‘we are all in this together.' Similar findings of increased empathic concern following economic threat, which in turn predicted prosociality, support this interpretation (Alonso-Ferres et al., 2020).

### Limitations

Methodological limitations of the present studies concern the validity of the measures employed and the generalizability of the findings to other populations. To assess prosociality, we measured willingness to donate and support for collective action, two central indicators of prosocial behavior (Louis et al., 2019). However, attitudinal and behavioral support for aid agencies was only assessed with one item, which could have weakened its validity. Similarly, to assess neoliberal beliefs, we selected eight items reflecting all sub-facets of the Neoliberal Beliefs Inventory (Bay-Cheng et al., 2015) but did not use the full scale. Future research may also other measures of neoliberalism that focus more on the psychological mindset accompanying the neoliberal ideology (Girerd et al., 2023).

Our samples consisted of young, primarily female and well-educated participants (most of whom had an upper secondary level of education) from German-speaking European countries. The findings may therefore be generalized to similar populations in Western industrialized countries. As neoliberalism was more prevalent among male participants in the present studies, this may have resulted in an underestimation of the effects of neoliberal beliefs on prosociality. However, the composition of the samples precludes such interference.

We found threat salience to motivate prosociality among neoliberals. However, we could only speculate about the underlying cognitive processes that drive these effects because we did not explicitly test them. Increased cognitive flexibility, norm salience, and group-based processes may help explain the present findings and need further investigation.

Social crises put shared social values and norms, such as individual responsibility, into question and can prompt individuals to reassess their ideological convictions. They have to reassess the situation, which requires cognitive flexibility. This could have made neoliberals in general more flexible in prosocial responses, but also more prone to act in line with salient prosocial values and norms.

Although we made the consequences of economic and humanitarian threats salient, we inferred only indirectly that this activated shared humanitarian values and prosocial norms. Future studies would benefit from directly manipulating norm salience by contrasting prosocial and individualistic norms in crisis contexts. Similarly, group-based processes should be tested more directly. While we found that threat salience increased identification with the outgroup, this effect may depend on the specific outgroup in need. Ukrainians may be perceived as more similar to the ingroup due to a shared European identity, whereas refugees from other regions may not elicit the same response.

### Other ideological convictions

Across studies, we found that the influence of neoliberalism on prosociality was independent of the effects of political orientation and SDO. While all three orientations correlated moderately positively with each other, they exerted an independent negative influence on prosociality. This is consistent with previous research and confirms neoliberalism as an independent normative worldview (Azevedo et al., 2019; Bay-Cheng et al., 2015; Beattie et al., 2019). Nevertheless, future research is needed to understand the interplay of threat salience with different system-stabilizing ideologies that are based in individualism (i.e., neoliberalism) and collectivism [i.e., belief in group superiority (SDO)].

## Conclusion

Neoliberal beliefs can be a barrier to social change and solidarity with those in need. However, this relation can be situationally challenged by salient crisis threats. Social crises that pose a threat to individual needs and shared values can make ideological convictions less important and counteract their detrimental effects on society. Together, they represent different motivational pathways to collective action (Radke et al., 2020). While people who endorse neoliberal beliefs may lack personal motivation to engage in collective action, threat salience can enhance outgroup motivation by strengthening social bonds with those in need. It may also activate moral motivation when humanitarian values are made salient in the situation.

## Data Availability

The datasets presented in this study can be found in online repositories. The names of the repository/repositories and accession number(s) can be found below: https://osf.io/qnfrt/?view_only=e3e2bfcf345b4cabab6e42446ecd03a4.

## Footnotes

Reviewed by: Kateryna Maltseva, National University of Kyiv-Mohyla Academy, Ukraine Alexander Vinogradov, Kyiv Institute of Modern Psychology and Psychotherapy, Ukraine
