# Supplementary material for: When neoliberals become activists: social crisis threats motivate ingroup and outgroup prosociality among neoliberals
Source: Front Psychol. 2025 Nov 7;16:1677265. doi: 10.3389/fpsyg.2025.1677265 (PMC12634635; doi:10.3389/fpsyg.2025.1677265)
Supplement: Supplementary file 1 [file Table_1.docx]

**Supplement**

**Table S1: Results of the moderated mediation analysis on solidarity-based action in Study 2.**

With threat salience as predictor, outgroup identification as mediator, neoliberal beliefs as moderator, and political orientation and SDO as covariates.

|  | ***coeff*** | ***SE*** | ***t(197)*** | ***p*** | ***95% CI*** |
| --- | --- | --- | --- | --- | --- |
| **DV: Outgroup Identification** |  |  |  |  |  |
| Constant | 4.31 | 0.26 | 16.53 | <.001 | [3.80, 4.83] |
| Threat Salience | 0.36 | 0.16 | 2.69 | .008 | [0.10, 0.63] |
| Threat x Neoliberal Beliefs | 0.19 | 0.12 | 1.54 | .125 | [-0.05, 0.43] |
| Neoliberal Beliefs | -0.24 | 0.10 | -2.50 | .013 | [-0.43, -0.05] |
| SDO | -0.07 | 0.08 | -0.89 | .373 | [-0.23, 0.09] |
| Political Orientation | -0.14 | 0.05 | -2.71 | .007 | [-0.23, -0.04] |
| **DV: Solidarity-based action** |  |  |  |  |  |
| Constant | 4.13 | 0.34 | 12.28 | <.001 | [3.46, 4.79] |
| Outgroup Identification | 0.49 | 0.06 | 8.12 | <.001 | [0.37, 0.61] |
| Threat Salience | 0.17 | 0.12 | 1.46 | .146 | [-0.06, 0.41] |
| SDO | -0.24 | 0.07 | -3.56 | .001 | [-0.37, -0.11] |
| Political orientation | -0.14 | 0.04 | -3.40 | .001 | [-0.22, -0.06] |
| **Conditional indirect effects** | **effect** | **BootSE** | **Boot 95% CI** | |  |
| High NLB (+1SD) | 0.28 | 0.11 | [0.07, 0.52] | |  |
| NLB (M) | 0.18 | 0.07 | [0.05, 0.33] | |  |
| Low NLB (-1SD) | 0.08 | 0.09 | [-0.09, 0.25] | |  |
